# The price of a drink: levels of consumption and price paid per unit of alcohol by Edinburgh's ill drinkers with a comparison to wider alcohol sales in Scotland

**DOI:** 10.1111/j.1360-0443.2010.03225.x

**Published:** 2011-04

**Authors:** Heather Black, Jan Gill, Jonathan Chick

**Affiliations:** Queen Margaret UniversityEdinburgh, UK

**Keywords:** Alcohol, legislation policy, minimum pricing, off-sales, policy, price, price/unit, purchase price, supermarket

## Abstract

**Aim:**

To compare alcohol purchasing and consumption by ill drinkers in Edinburgh with wider alcohol sales in Scotland.

**Design:**

Cross-sectional.

**Setting:**

Two hospitals in Edinburgh in 2008/09.

**Participants:**

A total of 377 patients with serious alcohol problems; two-thirds were in-patients with medical, surgical or psychiatric problems due to alcohol; one-third were out-patients.

**Measurements:**

Last week's or typical weekly consumption of alcohol: type, brand, units (1 UK unit 8 g ethanol), purchase place and price.

**Findings:**

Patients consumed mean 197.7 UK units/week. The mean price paid per unit was £0.43 (lowest £0.09/unit) (£1 = 1.6 US$ or 1.2€), which is below the mean unit price, £0.71 paid in Scotland in 2008. Of units consumed, 70.3% were sold at or below £0.40/unit (mid-range of price models proposed for minimum pricing legislation by the Scottish Government), and 83% at or below £0.50/unit proposed by the Chief Medical Officer of England. The lower the price paid per unit, the more units a patient consumed. A continuous increase in unit price from lower to higher social status, ranked according to the Scottish Index of Multiple Deprivation (based on postcode), was not seen; patients residing in postcodes in the mid-quintile paid the highest price per unit. Cheapness was quoted commonly as a reason for beverage choice; ciders, especially ‘white’ cider, and vodka were, at off-sales, cheapest per unit. Stealing alcohol or drinking alcohol substitutes was only very rarely reported.

**Conclusions:**

Because patients with serious alcohol problems tend to purchase very cheap alcohol, elimination of the cheapest sales by minimum price or other legislation might reduce their consumption. It is unknown whether proposed price legislation in Scotland will encourage patients with serious alcohol problems to start stealing alcohol or drinking substitutes or will reduce the recruitment of new drinkers with serious alcohol problems and produce predicted longer-term gains in health and social wellbeing.

## INTRODUCTION

In his 2009 annual report [1], the Chief Medical Officer for England predicted that there would be a reduction in health and social harms plus economic benefits if a minimum price for a unit of alcohol was set at 50 pence (£0.50). These predictions were based on models produced by Meier *et al*. (2009) and Purshouse *et al*. (2010), of the University of Sheffield [2,3]. The importance to health of setting a minimum price was made forcefully by Groves [4]. In Scotland, the current minority government has included minimum pricing as one of its proposed set of alcohol policies, but opposition parties to date have declined to accept this, partly on the grounds of lack of evidence that it would have the desired effects.

The literature on the elasticities of alcohol purchasing across several countries, reviewed in meta-analyses by Wagenaar *et al*. [5] and Gallet *et al*. [6], concluded a median elasticity of −0.51 and −0.497, respectively, implying that a 10% rise in price might be expected to reduce overall demand for alcohol by about 5%.

The University of Sheffield study used data on drinking and purchasing patterns drawn from the UK General Household Survey and the UK Expenditure and Food Survey to produce models to predict the effect that changing the purchase price of alcohol would have on the level of purchases or demand for alcohol by type of drinker, and also the ongoing effect that there might be on indices of harm. It was estimated that if alcohol prices increased, harmful drinkers (those defined as consuming alcohol at a level likely to affect their health adversely and/or cause other negative outcomes, i.e. in excess of 50 units per week for men and in excess of 35 units per week for women) would indeed reduce their overall consumption and give an estimate of the consequent decrease in alcohol-attributable hospital admissions and deaths.

One statement made in the Sheffield study [2] has been used by those who oppose minimum pricing: ‘—at the highest level of aggregation—hazardous and harmful drinkers (combined elasticity of −0.21) are less price elastic than moderate drinkers (elasticity of −0.47)’ (p. 58). Some took this to mean that when price is increased, hazardous and harmful drinkers might be more likely than light and moderate drinkers to switch to an alcoholic beverage which sold at a cheaper price per unit. However, the elasticities imply that if prices rose by 10%, although the heavy drinkers (e.g. 100 units/week) only reduce consumption by 2.1%, the absolute reduction of 21 units/week is a far greater reduction than that occurring among light/moderate drinkers (e.g. 10 units/week) of 0.47 units/week (see also discussion at the House of Commons Health Committee, 2010 [7]). It would seem helpful in this debate to also know whether harmful drinkers are already purchasing towards the very lowest end of the available price per unit range. It is on this point that our study is pertinent.

It should be noted that surveys such as the General Household Survey and the Expenditure and Food Survey, on which UK price–elasticity estimates are based, tend not to obtain data on the most extreme drinkers—those who are actually ill with their drinking—because such individuals may be hard to contact and if contacted are less likely to agree to participate [8].

This is recognized by many researchers as one reason why general population surveys of drinking account for only some 50% of national sales (e.g. Stockwell *et al*. [9] and Kerr & Greenfield [10]).

This study aims to contribute to the current debate by presenting empirical data on drinking among the heaviest consumers. To our knowledge, there is no UK study describing the purchasing habits of dependent and harmful drinkers. We have attempted to document this and view their purchasing habits in the wider context of Scottish drinking. We include data on a large number of women ill with alcohol. This is important, given that the greater prevalence of alcohol dependence in Scotland compared to England is due partly to the excess of females, the prevalence of alcohol dependence in females in Scotland being double that in England (3.3% versus 1.7%) [11].

## METHODS

We interviewed a sample of patients seen at two Edinburgh hospitals from September 2008 to June 2009 because of harm to health due to alcohol: those attending the Alcohol Problems Out-patient Clinic and in-patients at the detoxification and assessment ward, both at the Royal Edinburgh Hospital (REH), and in-patients admitted with medical and/or surgical problems to the Royal Infirmary Edinburgh (RIE) and referred to the Alcohol Liaison Service. They were asked to recall their most recent week of drinking (or if that was untypical or could not be recalled, a recent ‘typical week’) in terms of the types of drink consumed, volumes consumed (natural volume), alcoholic strength of drinks [percentage of alcohol by volume (ABV%)], brands of drinks (to enable accurate recording of ABV%), purchase price and where purchased. (In the United Kingdom, ‘on-sales’ means ‘sold on the premises of a bar, restaurant, club, pub or hotel’. Purchases from shops and supermarkets are termed ‘off-sales’.)

The interview would often last 30–60 minutes, discussion around volumes being the most time-consuming stage. On the whole, patients knew what volumes they had consumed in terms of cans/bottles and we had precise container volumes from the trade. Supermarket online shopping sites were used to check for pack sizes, e.g. if someone reported buying a box of cans on offer at a certain supermarket we used the internet to check strengths where there was uncertainty. On-licence measures in Scotland are standardized. For spirits, sometimes patients did not know whether their pub served 25 ml or 35 ml measures, in which case 25 ml was recorded; the patient would also advise if they had purchased a ‘single’ or ‘double’ measure. Sharing a bottle of wine, for example, with another drinker was not common, but in this case patients were asked to estimate what proportion of the bottle they consumed. Many had fixed drinking routines—same drinks/volumes each day. Some counted the empties the next morning. For wine, if patients did not know the strength it was recorded as 12.5%, which is in line with the Nielsen data that the Scottish government (SG) published [11], and is at the lower-end ABV sold these days in the United Kingdom. Strengths of vodka were recorded as 37.5% (except one or two people who advised differently), whisky was recorded as 40%—again in agreement with Nielsen/SG (see below).

From this information we calculated total alcohol units consumed per week and price paid per UK unit (8 g ethanol). Patients were also invited to offer reasons for their preferred alcohol purchasing habits.

Excluded were patients under 16 years old, those whose last week was not typical and who could not recall a period of their typical drinking which had occurred in the past 6 months, patients unable to read the information and consent form, and patients unable to understand English or with significant memory impairment due, for example, to Korsakov's dementia.

Also, patients being considered for liver transplant were not approached lest it interfere with the sensitive assessment and recommendation process. For logistical reasons every patient attending the alcohol services in this period could not be approached; however, data collection was continuous over the time-period. A total of 16 male and 25 female patients approached did not want to participate and staff declined participation on behalf of 18 male and eight female patients because the patient was too ill or was difficult and unlikely to cooperate. For 14 patients who were interviewed, the data could not be used for the analysis because the interview was stopped early or the responses were deemed unreliable. Interviews were recorded on an anonymized record sheet.

Socio-economic status is a possible confounder in any analysis of purchasing habits. Instead of asking relatively intrusive questions about income and employment category, we requested patients to provide their full postcode: the Scottish Index of Multiple Deprivation (SIMD) [12] allocates a score of deprivation to postcodes and we used the ranking of these scores as a proxy for income/social class.

## RESULTS

In all, 377 interviews could be used for analysis, comprising 256 men and 121 women (67.9% and 32.1%, respectively), whose mean age was 47 years (range 21–80) (47 for men and 46 for women). Of these, 30.0% were in-patients at RIE, 37.9% were in-patients at REH and the remaining 32.1% were out-patients at REH.

**Figure 1:**
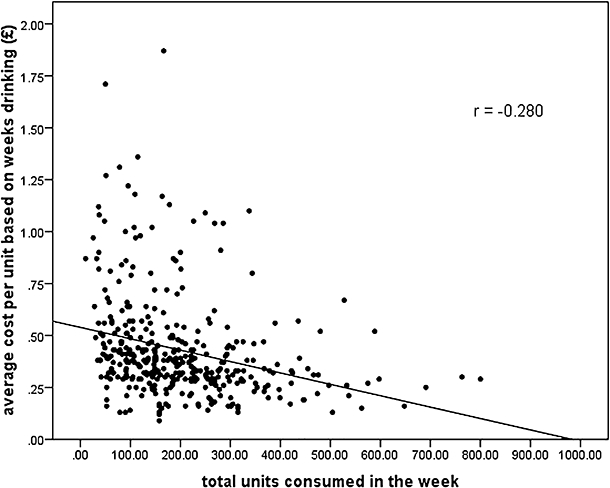
Scattergraph showing correlation between unit price paid (£) and units consumed in the most recent week of drinking for all patients

Table 1 lists the mean number of units consumed in the week and the mean price paid per unit for all patients and by type of purchase (on-sales and off-sales). For all alcohol intake (on-sales and off-sales), the mean number of units consumed for all patients was 197.7 [range 10.3–800.2; 95% confidence interval (CI) 184.8–210.7]. The mean price paid per unit for all alcohol was £0.43 (range 0.09–1.87; 95% CI, 0.41–0.46; £1.00 = US$1.6 or €1.2). The mean price paid by each patient is plotted as a function of total units consumed in the week for each patient in Fig. 1.

**Table 1:** Patients' purchasing by on-sales and off-sales and price paid per unit

*Type of purchase*	*Mean units consumed per week (range) (95% CI)*	*Mean price (£) per unit (range) (95% CI)*	*Percentage of their week's units consumed purchased from this source (range) (95%CI)*
Purchased some as on-sales (*n* = 96 patients)	71.3 (2.3–292.3) (56.5–86.1)	1.1 (0.59–2.37) (1.04–1.18)	46.5 (0.35–100) (39.1–53.9)
Purchased some as off–sales (*n* = 359 patients)	188.7 (4.2–800.2) (175.2–202.2)	0.34 (0.09–1.03) (0.33–0.36)	92.6 (5.4–100) (90.6–94.6)

CI: confidence interval.

With decreasing unit price, a patient's mean unit consumption increases (Fig. 1; *r* = −0.280, *P* < 0.01), especially with respect to off-sales (*r* = −0.34, *P* < 0.01).

A total of 96 patients (25%) consumed any of their units as on-sales (i.e. in pubs, hotels, restaurants, bars and clubs), but this accounted for only 7.4% of the total units that those drinkers consumed.

Of drinks purchased as off-sales, almost all were purchased from supermarkets, or from local shops/licensed grocers, in equal proportions. Of the units consumed, 70.3% of all units were sold at or below 40p (£0.40), and 82.6% were sold at or below 50p (£0.50).

No patients reported illegal purchasing of beverages or consuming illicitly produced alcohol. One patient reported consuming a very small amount of substitute alcohol in the form of perfume, in addition to purchased drinks. The ‘other’ category includes off-sales drinks which were purchased by someone else and stolen drinks: four patients reported stealing alcohol (three from a supermarket, one from a licensed grocer), which amounted to approximately 0.4% of the sum of units they each purchased as off-sales for the week (mean/patient 284 units).

When asked an open-ended question about the reasons for their alcohol purchasing habits, cheapness was mentioned by 47.5% of patients; among those spending £0.40 or less per unit, cheapness was mentioned by 60.8% (141 of 232). However, for white ciders (the cheapest drinks), 61 of the 66 patients chose it because it was cheap (some said ‘cheap and strong’), the five others said they bought it because of its strength. These were the only two reasons ever given for white cider purchase.

As expected, compared to the wider population, a higher proportion of patients resided in the lower quintiles (quintile 1 comprises those postcodes with the greatest deprivation, quintile 5 those with least deprivation [12]), with just over 50% belonging to quintiles 1 and 2 (the expected proportion in each quintile is 20%).

There was no difference between the SIMD quintiles in terms of mean expenditure per unit (one-way analysis of variance, ANOVA) (Table 2). Although the lowest overall unit price of £0.09/unit was purchased by a resident of the highest quintile, patients from quintile 1 paid the lowest mean unit price (mean £0.30), consumed the largest amounts purchased from off-sales (mean 224.3 units/week) and spent the most (mean £82.36/week), the mean expenditure per week for all patients being £75.57. Some other patients also found a £0.09/unit source by buying packs of 2-litre bottles of white cider at supermarkets or ‘cash-and-carry’, but consumed some other beverages as well which increased their mean price per unit. (As an aside, the sum of the expenditure for this sample of 377 patients for one week's alcohol consumption was £28 491.)

**Table 2:** Consumption by SIMD quintile

*SIMD quintile*	*n*	*Percentage of total*	*Mean week's consumption (units) (range) (95% CI)*	*Mean price paid per UK unit (£) (range) (95% CI)*	*Mean % of units purchased as off-sales (95%CI)*
1 (lowest SES)	107	28.4	228.8 (10.3–800.0) (199.0–258.7)	0.400.13–1.27) (.35–0.45)	88.0 (82–94)
2	88	23.3	200.4 (32.5–575.9) (174.0–226.9)	0.39 (0.12–1.16) (0.35–0.43)	93.4 (89–97)
3	61	18.2	184.3 (25.6–691.0) (150.0–218.8)	0.53 (0.15–1.71) (0.44– 0.62)	74.9 (65–84)
4	52	12.8	157.3 (36.0–447.4) (133.3–181.2)	0.43 (0.14–1.02) (0.35–0.49)	89.8 (83–97)
5	69	17.3	188.4 (28.1–393.8) (166.4–210.5)	0.44 (0.09–1.87) (0.38–0.40)	92.3 (87–97)
Overall	377			0.43 (0.09–1.87) (0.40–0.47)	88.1 (85–91)

SIMD: Scottish Index of Multiple Deprivation; SES: socio-economic status.

An ANOVA followed by *post-hoc* tests revealed a statistically significant difference in the mean unit price paid for all alcohol by those patients in quintile 3 (£0.53) compared with those patients in quintiles 1 and 2 who reported mean unit prices of £0.40 and £0.39, respectively. However, there is not a continuous increase in mean unit price from the lowest to the highest quintile. This appears to be partly because patients from higher social groups, like those from lower social groups, also purchase much of their alcohol from off-sales. Further analysis showed that those in quintile 3 (*n* = 61) purchased 23.1% of their units as on-sales compared to the total sample mean (*n* = 377) of 11.9%.

For the entire patient sample, an independent-samples *t*-test showed a significant difference between the percentage of units consumed by men and women as on-sales (mean percentage for men was 14.5% and for women was 6.3%, *P* = 0.02, equal variance not assumed). Thus, a possible reason why patients from quintile 3 consume proportionately more as on-sales was due to the slightly higher proportion of men in quintile 3 (in this sample), 75.4% compared to the total sample mean of 67.9%. However, a χ^2^ test of gender by quintile found this not to be statistically significant. An ANOVA test was performed to check whether age may be a factor in the quintile difference for on-sales consumption, but the results did not reveal a significant difference in mean age between the quintiles.

### 

#### The heaviest consumers

A total of 137 individuals consumed 200 units or more per week, drinking a mean of 299 units/week. The majority (60.5%) were in quintiles 1 and 2, although 19% belonged to quintile 5.

These heaviest consumers paid a mean of £0.30 per unit (range 0.13–0.58) in contrast to those who drank less than 200 units/week who paid a mean of £0.37 (range 9–103) and purchased 97% of their alcohol from off-sales in contrast to 89%. A one-way analysis of variance performed on the group consuming 200 units or more per week did not show a statistically significant difference in the mean unit price between the quintiles (*P* = 0.55).

#### Comparison to wider Scottish alcohol sales

We obtained national sales data for 2008 from The Nielsen Company. The pattern of beverage types consumed by patients is different from that of the wider population when their week's pattern, in terms of units of alcohol, is compared to national sales figures (calculated after excluding those drinks, such as champagne, never or very rarely consumed by patients). Vodka is the patients' most popular drink (28.6% of their total units purchased versus 13.1% purchased by the wide population for that year). Lager and beer are the most popular among the wider population (36.9% versus 13.4%), although within that, super strength lager/beer is rarely drunk by the wider population (0.006% of their total units) but accounts for 7.8% of patients' total units. The most striking difference is that white cider accounts for 16.0% of patients' week's consumption but is a rare preference (0.009%) for the wider population. Other ciders account for 8.0% of patients' and 0.09% of the wider population's consumption.

The mean unit price paid at purchase in Scotland in 2008 for all sales of alcohol was £0.71, according to sales data (till receipts) collected by The Nielsen Company and published by Health Scotland [13]. Excluding the categories of beverage rarely if ever consumed by our patients, the price per unit for the wider population is £0.67. From the detailed sales data we obtained from The Nielsen Company for 2008, we could compare the prices paid for each class of beverage. Table 3 suggests that a main factor in the difference between patients' somewhat cheaper purchasing than the wider population, shown in Table 3, is that patients purchase a greater proportion of their alcohol off-licence than the wider population (93% compared to 69%). It is noteworthy that 75% of patients never purchased alcohol from on-sales settings.

**Table 3:** Unit price paid by patients, and % less than the wider Scottish drinkers' purchase price in 2008

	*All sales*				*Off-licence sales*			
		
	*Patient n*	*Patient unit price £*	*Scottish unit price*	*Patient price relative to Scottish price as %*	*Patient n*	*Patient unit price £*	*Scottish unit price*	*Patient price relative to Scottish price as %*
Vodka	158	0.39	0.58	−32.76	149	0.33	0.34	−2.94
Whisky	55	0.59	0.60	−1.67	44	0.34	0.41	−16.07
Brandy	10	0.50	0.63	−20.63	10	0.39	0.37	+5.41
Gin	12	0.55	0.64	−14.06	9	0.43	0.43	0.00
White rum	4	0.48	0.74	−35.14	4	0.35	0.40	−13.50
Dark rum	7	0.36	0.87	−58.62	7	0.36	0.42	−14.29
Super lager or beer	39	0.34	0.34	0.00	39	0.34	0.32	+6.25
Strong lager or beer	57	0.51	0.62	−16.74	46	0.35	0.39	−10.26
Lager or beer	119	0.67	0.85	−21.17	78	0.39	0.39	0.00
White cider	66	0.15	0.17	−18.67	66	0.15	0.17	−18.67
Strong cider > 6%	13	0.30	0.33	−9.09	13	0.30	0.21	+42.86
Cider < 6%	56	0.54	0.65	−18.92	48	0.34	0.37	−8.11
White wine	67	0.55	0.60	−8.33	64	0.46	0.44	+4.55
Red wine	28	0.48	0.60	−20.00	27	0.47	0.45	+4.44
Fortified wine	23	0.40	0.49	−17.37	23	0.40	0.40	0.00
Overall	377_a_	0.46	0.67_c_	−20.0	359_b_	0.34	0.40	−15.0

Some drinkers purchased more than one type of beverage so *n* columns do not add up to 377^a^ and 359^b^, respectively. ^c^The Scottish unit price is very slightly less than the overall unit price for Scotland 2008 of £0.71 [13] because certain expensive categories of beverage, e.g. champagne, are not included in this table due to being rarely, if ever, reported by patients.

When their off-licence purchasing is compared with wider off-licence sales (Table 3), of their five most popular beverages (in order: vodka, ordinary lager/beer, white cider, white wine, cider < 6% and whisky) it is only whisky and white cider that they purchased off-licence significantly more cheaply than the wider population. (‘Ready-to-drink’ beverages, champagne, sparkling wines and liqueurs are not included in Table 3 because these contributed extremely little to the total reported by patients.)

Caffeine (e.g. in ‘energy drinks’) and alcohol are sometimes consumed together. A beverage claimed by some to be especially problematic in Scotland is ‘Buckfast’ caffeine-containing wine. Twelve patients (3.18%) reported drinking it in the week, ranging from 0.56 l to 10.5 l per individual (mean 3 l/drinker/week), at a mean unit price of £0.51.

## DISCUSSION

The advantages to society of imposing a minimum unit price for alcohol, from the perspective of public health and social harms, depend on reducing recruitment to heavy drinking and on reducing the consumption of those drinkers who already contribute to alcohol harm statistics or are at high risk of doing so. [Since this work was completed, the Scottish government has decided to propose a minimum price of £0.45 per unit but legislation is not yet passed.] The patients in our study had all experienced such harm and sometimes caused harm to others. We have presented evidence that demonstrates:

- that this patient population purchases alcohol units on average at £0.29 less per unit than that paid on average by the general Scottish population, that their purchases are mainly in off-sales, that this is not simply explained by social status, and that within the off-sales market they buy at £0.06/unit lower than the average Scottish off-sales purchase. Kerr & Greenfield [10] similarly found for the general population of the United States that lower expenditures per drink by the heaviest drinkers suggest substantial downward quality substitution, drinking in cheaper contexts and other bargain pricing strategies;
- that of these patients, those who pay the lowest prices per unit tend to consume the greatest number of units; and
- that this patient population purchase 83% of their alcohol at or below £0.50/unit.

Our sample does not represent all harmful drinkers in Scotland. The exclusion criteria applied to the study meant that all Edinburgh patients who contribute to both the alcohol harm statistics and alcohol sales data in Scotland could not be approached for interview. Those too ill to participate, patients awaiting liver transplant and those under 18 years of age (a group who may be especially liable to purchase cheaply) and ill drinkers who do not access services are not represented in our data. We had to exclude those with poor communication and literacy skills who may be some of those whose first language is not English. The characteristics of this un-sampled group will vary by region within Scotland, and so our results cannot be assumed necessarily to apply throughout the country.

It can be argued that SIMD as a proxy for social status does not deal effectively with the possibility that buying cheaply is simply a feature of the lower social status of our sample. Nevertheless, we found, for each SIMD quintile, a statistically significant correlation between decreasing unit price and increasing consumption and, perhaps surprisingly, this appeared most strongly in the high social status quintile, quintile 5. Indeed, the lowest prices being paid per unit (£0.09) by any patient in the study was a patient in quintile 5. The mean price per unit paid by our patient sample for off-sales alcohol for each of the SIMD quintiles (range £0.31–0.38) is less than the mean for off-sales in Scotland in 2007 of £0.40 [13].

Postcode might be an unreliable proxy for social class if a patient was still living with his family of origin in a postcode above his current ‘fallen’ social status, but previous studies in our clinics found it unusual for a patient to still live with family of origin. Although a few patients might have given a hostel as their address, such hostels in Lothian tend to be located in areas with low SIMD ranks, and that would not distort our use of postcode data.

Another finding which encourages us to believe that our use of SMID is not an unreliable proxy is that the UK Food and Expenditure Survey shows that purchasing alcohol cheaply (less than £0.31/unit) is spread surprisingly evenly across all households irrespective of income, except for the highest 10% of households [14]. Thus, we do not believe our finding of extensive cheap alcohol purchasing by our patients is due particularly to their low social status or income.

Whether setting a minimum unit price in Scotland of, for instance, £0.40 or £0.50, or other devices to reduce cheap discounting and low price sales would reduce the units consumed by drinkers such as the patients presented here cannot be answered definitively without monitoring consumption trends should such policies be enacted. However, as a response to criticism of elasticity models, our findings suggest that hazardous and harmful drinkers have little ‘room’ for elasticity as they already purchase their alcohol at very low prices, and indeed shop around to do so. The point made by the Centre for Business and Economic Research [15] about the Sheffield price elasticity calculations that ‘the evidence seems to suggest that heavier drinkers are more likely to switch from one product category to another in the face of price changes' (p. 22) is made less relevant by our findings for two reasons: first, because the ‘heavier drinkers’ in the elasticity computations derived from survey data are not nearly such heavy drinkers as our patients (the General Household Survey which was used in the analysis by Meier *et al*. [2] defined harmful and hazardous drinkers as consuming in excess of 50 or 35 units per week, respectively, for men and women, whereas our patients consumed a mean of almost 200 units with a range up to 800 units/week); and secondly, because we found that many of the seriously harmed drinkers were already drinking as cheaply as they could—if minimum pricing was enacted, the cheapest brand of, for instance, cider would cost the same per unit as the cheapest brand of lager, and so those drinkers who want the most ethanol for their pennies would have no monetary incentive to switch.

We suggest that in such very heavy consumers, a small percentage change in purchasing can be expected to have a relatively large absolute effect on their consumption. Of course, supposing this population had no financial constraint, if prices rose they would continue to buy as much as they wanted. We did not collect data on personal finances and cannot rule out that possibility, although some patients reported that they ceased their week's purchasing of alcohol because their weekly funds were finished.

Another objection to minimum pricing as a public health strategy is that ‘dependent’ drinkers will turn to substitute or illicitly distilled alcohols, or steal alcohol. While this may fit a caricature of the alcoholic, we obtained almost no reports of such behaviour in these 377 patients, many of whom would have met criteria for ‘dependence’. A considerable shift in self-concept of this population would have to occur for substantial numbers to fulfil that stereotype. Again, if pricing legislation is implemented only a further study can respond conclusively to that objection.

## FURTHER IMPLICATIONS

The minimum price paid in the on-sales setting was £0.59/unit, i.e. a value in excess of one of the minimum price levels (£0.50/unit) currently being considered in the United Kingdom. Our clinical experience, acquired unsystematically from drinkers' self-reports, suggests that it is sometimes preferable for a dependent drinker to drink in a pub or club where there are external controls on the amount consumed and the level of intoxication and also possible mental health benefits of social interaction. Minimum pricing legislation might increase the on-sales proportion of the consumption of dependent drinkers.

Although there was a plan in 2010, now reversed by the incoming government, that excise tax on cider in the United Kingdom should be brought nearer to the excise tax on other alcoholic beverages of similar strengths, it appears that duty increases can be absorbed readily by the industry or supermarkets. The makers of a well-known brand of cider even advertised their intention to absorb the proposed rise (‘Same again Darling. We're covering the cost of the duty increase’[18]). In fact, the small rise proposed in excise on cider would have had only a small impact even without absorption by the industry: for example, a 2-litre bottle of cider costing £1.20 would rise to £1.32. This still works out at only 15p per unit of alcohol (prices checked on day of original announcement, March 2010 [16]).

So-called ‘white cider’, the source of the lowest price per unit for many of our heaviest drinkers, has a lower concentration of anti-oxidants than cider made directly from apples, and this has been postulated to make it a relatively harmful beverage compared to beverages with higher anti-oxidant content [17].

For Scotland, a pressing concern is the growing prevalence of alcohol problems among women, double that in England [11]. Female patients on average paid a lower unit price than males (£0.41 versus £0.44) and purchased less as on-sales than men (6.3% of their units as on-sales, compared to 14.5%). Pricing changes, because they will affect almost exclusively off-sales, may help towards alleviating alcohol problems among Scotland's women.
